# Acupuncture treatment for emotional problems in infertile women: A protocol for systematic review and meta-analysis

**DOI:** 10.1097/MD.0000000000026306

**Published:** 2021-06-11

**Authors:** Su-In Hwang, Soo-Hyun Sung, Young-Jin Yoon, Jang-Kyung Park

**Affiliations:** a Department of Korean Medicine Obstetrics and Gynecology, Pusan National University Korean Medicine Hospital; b Department of Policy Development, National Development Institute of Korean Medicine; c Division of Clinical Medicine, School of Korean Medicine, Pusan National University, Yangsan, South Korea.

**Keywords:** acupuncture, infertile women, infertility, infertility-related emotional problems, meta-analysis, protocol, systematic review

## Abstract

**Background:**

Infertility causes emotional and psychological problems, including anxiety, depression, low self-efficacy, and chronic mental stress in women. These emotional problems can negatively affect fertility treatment. Numerous studies have reported the clinical therapeutic effects of acupuncture on emotional problems; however, the efficacy and safety of acupuncture treatment for emotional problems in infertile women remain unclear. This protocol aims to evaluate the efficacy and safety of acupuncture for treating emotional problems in infertile women.

**Methods:**

We will search the following databases from their inception to April 30, 2021: MEDLINE, EMBASE, Cochrane Library, Korean Medical Databases (KoreaMed, Korean studies Information Service System, Korean Traditional Knowledge Portal, Oriental Medicine Advanced Searching Integrated System, Research Information Sharing Service, and National Digital Science Library), and Chinese databases (CNKI and Wan Fang Database). We will include randomized controlled trials on acupuncture for emotional problems in infertile women. There will be no restrictions regarding language or publication date. The primary outcome will be assessed using an emotion-related assessment scale. The risk of bias of the included studies will be measured using the Cochrane risk of bias assessment tool. For meta-analysis, RevMan Version 5.4 software will be used to combine the relative risks for dichotomous outcomes, as well as the mean differences or standardized mean differences for continuous outcomes, with both having 95% confidence intervals.

**Results:**

Based on current evidence, this study will assess the effectiveness and safety of acupuncture for emotional problems in infertile women.

**Conclusion:**

This study will provide evidence for evaluating the acupuncture efficacy for infertile women with emotional problems.

**Registration number:**

INPLASY202150082.

## 1 Introduction

The World Health Organization defines infertility as the failure to establish a clinical pregnancy after 12 months of regular unprotected sexual intercourse.^[1]^ Worldwide, 15% of reproductive-aged couples are estimated to be infertile.^[2]^ There has been a recent increase in the infertility prevalence due to various factors, including marital status, educational attainment, unfavorable lifestyle, increased reproductive diseases, artificial abortion, long-term contraceptive use, and increased environmental pollution.^[3,4]^

Infertility causes emotional and psychological problems in women,^[5]^ including anxiety, depression, and low self-efficacy.^[6]^ These emotional problems can cause emotional distress, as well as negatively affect fertility treatment, which results in treatment discontinuation and a decrease in the pregnancy rate.^[7,8]^

To diminish negative emotional symptoms and achieve higher pregnancy rates, many infertile women have been exploring alternative therapies and psychological interventions, including psychoanalytic interventions, relaxation, cognitive behavioral therapies, and online counseling.^[9]^ Acupuncture is widely used in Eastern Asia as a key remedy for psychological problems.^[10]^ Previous studies have reported that acupuncture is effective for improving emotional symptoms, including anxiety,^[11]^ depression,^[12,13]^ low self-efficacy,^[14]^ and chronic mental stress,^[15]^ which could indicate a therapeutic effect on improving emotional symptoms in infertile women. Despite the extensive research on the therapeutic effect of combining acupuncture and in vitro fertilization to improve pregnancy and implantation rates,^[16–19]^ the acupuncture effect on improving the emotional problems in infertile women remains unclear. A systematic review of acupuncture treatment for emotional problems in infertile women was published in 2007^[20]^; however, it only addressed anxiety among infertile women without addressing other emotional problems.

Therefore, we intend to perform a systematic review to evaluate the effectiveness and safety of acupuncture treatment for managing emotional problems in infertile women.

## 2 Methods

### 2.1 Study registration

The study protocol has been registered in INPLASY, an International Platform of Registered Systematic Review and Meta-analysis Protocols (https://inplasy.com/inplasy-2021-5-0082/) and INPLASY registration number is INPLASY202150082. This protocol was written in accordance with the Preferred Reporting Items for Systematic Reviews and Meta-analysis Protocols (PRISMA-P) guidelines.^[21]^

### 2.2 Eligibility criteria for study selection

#### 2.2.1 Types of studies

All randomized controlled trials (RCTs) evaluating the acupuncture treatment effect on emotional problems in infertile women will be included. Other studies, including non-RCTs, case series, case reports, crossover studies, letters, or laboratory studies, will be excluded. Study eligibility will not be restricted by language or publication date.

#### 2.2.2 Types of participants

This study will include women diagnosed with infertility and emotional problems. These emotional problems include anxiety, depression, low self-efficacy, distress, fear, panic, and nervousness. There will be no restrictions on age, race, nationality, education, or economic status.

#### 2.2.3 Types of interventions

Any acupuncture type will be accepted, including acupuncture, acupressure, electroacupuncture, auricular acupuncture, scalp acupuncture, hand acupuncture, pharmacopuncture, and transcutaneous electric acupoints. Control interventions will include placebo/sham acupuncture, no treatment, or conventional treatment. RCTs on the combined effects of acupuncture and conventional treatment will be included if identical conventional treatment was applied to both groups.

#### 2.2.4 Types of comparisons

The following treatment comparisons will be considered.

(1) Acupuncture versus placebo/sham acupuncture.
(2) Acupuncture versus no treatment.
(3) Acupuncture versus conventional treatment.
(4) Acupuncture plus conventional treatment versus identical conventional treatment alone.

#### 2.2.5 Types of outcome measures

##### 2.2.5.1 Primary outcomes

Emotion-related assessment scales (e.g., Hamilton Anxiety-rating Scale, State–trait Anxiety Inventory, Amsterdam Preoperative Anxiety and Information Scale, Self-rating Depression Scale, The Fertility Problem Inventory, Perceived Stress Scale, and Infertility Self-Efficacy scale).

##### 2.2.5.2 Secondary outcomes

(1) Total effectiveness rate for emotional problems.
(2) Quality of life.
(3) Clinical pregnancy rate.
(4) Adverse events.

### 2.3 Search methods for identification of studies

#### 2.3.1 Data sources

The following electronic databases will be searched from their inception to April 30, 2021: MEDLINE, EMBASE, Cochrane Library, Korean Medical Databases (KoreaMed, Korean studies Information Service System, Korean Traditional Knowledge Portal, Oriental Medicine Advanced Searching Integrated System, Research Information Sharing Service, and National Digital Science Library), and Chinese databases (CNKI and Wan Fang Database).

#### 2.3.2 Search strategy

The search terms to be used are (“infertility” OR “subfertility” OR “subfertile” OR “oligospermia” OR “azoospermia” OR “obstructive azoospermia” OR “genital disease”) AND (“emotion(s)” OR “emotional” OR “mood(s)” OR “feeling(s)” OR “psychological” OR “personality” OR “anxiety” OR “anxious” OR “anxiousness” OR “depression” OR “depressive” OR “stress” OR “distress” OR “distressing” OR “pain(s)” OR “painful” OR “fear(s)” OR “panic(s)” OR “nervousness” OR “self-efficacy” OR “relaxation” OR “adaptation” OR “mental disorder”) AND (“acupuncture” OR “acupressure” OR “electroacupuncture” OR “auricular acupuncture” OR “scalp acupuncture” OR “hand acupuncture” OR “pharmacopuncture” OR “transcutaneous electric acupoint”) AND (“randomized controlled trial” OR “randomized clinical trial”).

### 2.4 Data collection and analysis

#### 2.4.1 Selection of studies

**Figure 1:**
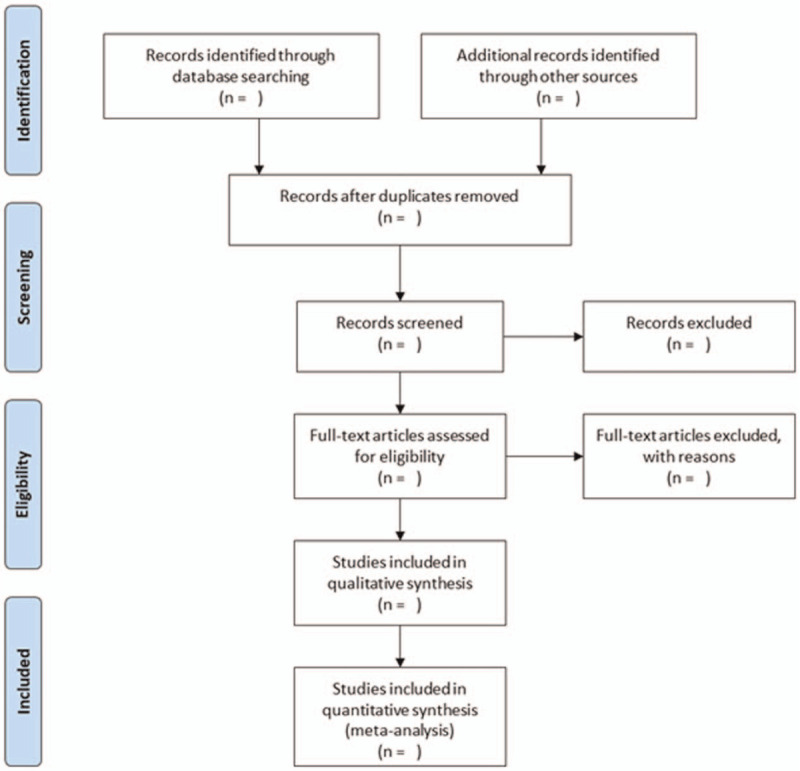
PRISMA flow diagram of the study process. PRISMA = Preferred Reporting Items for Systematic Reviews and Meta-Analysis.

Two authors will independently review and screen the titles and abstracts of included studies using pre-determined criteria to identify potentially eligible studies. Disagreements will be resolved by discussion or arbitrated by a third author. The flow of the study selection process will be based on the PRISMA flow diagram (Fig. 1).^[22]^

#### 2.4.2 Data extraction

Using a pre-standardized data extraction form, 2 independent reviewers will extract data regarding the authors’ information, participants, types of emotional problems, randomization, interventions (e.g., acupuncture type, acupuncture point, needle type, insertion depth, insertion angle, needle retention time, treatment period, treatment frequency), outcomes, and number of treatment-related adverse events. Details regarding the acupuncture treatment and control interventions will be extracted based on the revised Standards for Reporting Interventions in Clinical Trials of Acupuncture.^[23]^ Disagreement on extraction will be resolved through discussion with a third author.

#### 2.4.3 Assessment of risk of bias in the included studies

Two review authors will independently evaluate the risk of bias using the Cochrane risk of bias assessment tool.^[24]^ The following domains will be assessed: random sequence generation, allocation concealment, blinding of participants, blinding of outcome assessors, incomplete outcome data, selective outcome reporting, and other sources of bias (including factors likely to influence the results, such as extreme baseline imbalance of age, comorbidity, onset, or physical conditions). The risk of bias will be classified into 3 levels: low, high, and unclear. Disagreements will be resolved by reaching consensus with a third reviewer.

#### 2.4.4 Data synthesis

For meta-analysis, RevMan Version 5.4 software (The Cochrane Collaboration, 2020) will be used to combine the relative risks for dichotomous outcomes and mean differences or standardized mean differences for continuous outcomes, with both having 95% confidence intervals. We will pool data across the studies for meta-analysis using random-effect or fixed-effect models.

#### 2.4.5 Managing missing data

In case of missing or incomplete information, we will attempt to contact the first or corresponding author by email requesting adequate information. If the data cannot be obtained, we will perform analysis using the available data. However, the potential impact of the missing data will be considered and addressed in the discussion section.

#### 2.4.6 Assessment of heterogeneity

Based on the Cochrane Handbook for Systematic Reviews of Interventions,^[25]^ we will test data heterogeneity using a standardized chi-squared test and calculate the *I*^2^ statistics. If *I*^2^ < 50%, between-study statistical heterogeneity can be ignored and a fixed-effect model will be used for statistics. If *I*^2^ > 50%, between-study heterogeneity will be considered statistically significant and the random-effect model will be used. In case of considerable heterogeneity, we will conduct a subgroup analysis to identify the heterogeneity source.

#### 2.4.7 Subgroup analysis

If data are available, subgroup analysis will be conducted based on the types of acupuncture treatment (e.g., acupuncture, electroacupuncture, pharmacopuncture, or scalp acupuncture) and emotional problems.

#### 2.4.8 Assessment of reporting bias

We will use a funnel plot to evaluate the reporting bias when the number of studies included in the meta-analysis exceeds 10.^[26]^

#### 2.4.9 Sensitivity analysis

Sensitivity analysis will be performed to determine the robustness of the review results with respect to the following aspects: impact of sample size, effect of missing data, and methodological quality.^[27]^

#### 2.4.10 Grading the quality of evidence

Grading of Recommendations Assessment, Development, and Evaluation will be used to evaluate the quality of evidence for key outcomes. The quality of evidence will be graded into 1 of 4 levels: high, moderate, low, and very low.^[28]^

### 2.5 Ethics and dissemination

Ethical approval is not necessary since this systematic review will be based on published research. The results of this review will be disseminated through peer-reviewed journal articles and conference presentations.

## 3 Discussion

Infertility is an important public health issue that affects childbearing couples worldwide. It can cause marital instability, as well as increase depression, anxiety, low self-efficacy, and other emotional problems.^[29,30]^ Moreover, psychological pain related to infertility can adversely affect the outcome of fertility treatments.^[31]^ There is strong evidence supporting the effectiveness and safety of acupuncture for emotional problems, including anxiety, depression, low self-efficacy, and chronic mental stress. However, it remains unclear whether acupuncture is effective and safe for the emotional problems related to infertility given the lack of previous studies and systematic reviews on emotional problems in infertile women.

This paper presents a study protocol of a systematic review and meta-analysis of the use of acupuncture therapy for treating emotional problems in infertile women. Based on this protocol, we will perform a systematic review and meta-analysis to obtain evidence regarding the effectiveness and safety of acupuncture treatment for emotional problems in infertile women.

## Author contributions

**Conceptualization:** Su-In Hwang, Jang-Kyung Park.

**Funding acquisition:** Jang-Kyung Park.

**Investigation:** Su-In Hwang, Young-Jin Yoon.

**Methodology:** Su-In Hwang, Soo-Hyun Sung, Jang-Kyung Park.

**Supervision:** Jang-Kyung Park.

**Writing – original draft:** Su-In Hwang.

**Writing – review & editing:** Soo-Hyun Sung, Young-Jin Yoon, Jang-Kyung Park.
